# Delegates to the National Medical Convention

**Published:** 1847-01

**Authors:** 


### RECORD OF MEDICAL SCIENCE.

*Delegates to the National Medical Convention.*—The Medical Faculty of the University of Pennsylvania, have appointed of their number, Drs. Chapman, Jackson, and Wood, to represent them in the Convention in May next.

The Philadelphia Medical Society, at a meeting held November 21st, elected the following gentlemen as delegates to the Convention: Drs. Bell, Emerson, Isaac Parrish, West, Ashmead, Norris, B. H. Coates, Bond, S. G. Morton, Yardley, and Griscom. There is still a vacancy to be filled up by the election of a delegate, so as to complete the number, twelve, authorised by the Society.

The College of Physicians of Philadelphia, at a meeting, Dec. 1st. appointed a delegation to consist of Drs. Hewson, J. W. Moore, S. Jackson (formerly of Northumberland County), Hays, A. Stillé, J. R. Paul, Pepper, Fox, Randolph, C. Morris, Condie, and Bridges.

The Medical Society of New Jersey, which dates its origin from the 23d of July, 1766, and to age adds the merit of high standing among similar associations in the United States has elected delegates to the Convention. The list consists of Drs. Smith and Peirson of Essex County, Marsh of Passaic, Stewart of Morris, Forman of Mercer, Parrish of Burlington, Taylor and Cooper of Camden, Garrison of Gloucester, and Howell of ——.

The District Medical Society of Burlington County has appointed, as delegates, Drs, Cole, Stratton, and Read.

The delegates of the Philadelphia Medical Society, and of the College of Physicians, have been directed, respectively, to make arrangements, in connexion with the delegates that may be appointed by other institutions in the city, for the reception and a suitable place of meeting of the Convention.

The Vermont Medical Society has appointed Drs. Charles Hall, C. W. Horton, A. G. Dana, and Dyer Storer, as its delegates to the Convention.—*Bulletin of Medical Science.*

